# Comparison of Tissue-Engineered Dermis with Micronized Adipose Tissue and Artificial Dermis for Facial Reconstruction Following Skin Cancer Resection

**DOI:** 10.3390/bioengineering12020145

**Published:** 2025-02-03

**Authors:** Kyu-Il Lee, Won-Seok Song, Seung-Kyu Han, Kyung-Chul Moon, Seong-Ho Jeong, Eun-Sang Dhong

**Affiliations:** 1 Department of Plastic Surgery, Taean Public Health Office, Taean-gun 32148, Republic of Korea; 2 Department of Plastic Surgery, Korea University College of Medicine, Seoul 02841, Republic of Korea; 3 Department of Plastic Surgery, Korea University Guro Hospital, 148 Gurodong-Gil, Guro-Ku, Seoul 08308, Republic of Korea

**Keywords:** wound repair, biomaterials, tissue-engineered dermis, micronized adipose tissue

## Abstract

Our group has previously demonstrated that tissue-engineered dermis containing cultured fibroblasts or adipose-derived stromal vascular fraction cells is superior to artificial dermis in terms of scar quality for covering facial defects. However, using these cells for clinical applications requires Food and Drug Administration approval and involves complex procedures for cell culture or isolation. This retrospective study aimed to compare effects of tissue-engineered dermis containing micronized adipose tissue (MAT) and artificial dermis for facial reconstruction. Tissue-engineered dermis consisting of MAT seeded on artificial dermis was applied in 30 cases, while artificial dermis without MAT was grafted in 35 cases. Healing time and severities of scar contraction, color mismatch, and landmark distortion at one year after healing were evaluated. Wounds in the tissue-engineered dermis group re-epithelialized in 30.0 ± 4.3 days compared to 34.3 ± 5.4 days in the artificial dermis group (*p* < 0.05). The average dE2000 score in color mismatch analysis was 4.9 ± 1.7 in the tissue-engineered dermis group and 5.1 ± 1.7 in the artificial dermis group (*p* = 0.57). The extent of scar contraction was 16.2 ± 12.3% in the tissue-engineered dermis group and 23.2 ± 12.8% in the artificial dermis group (*p* < 0.05). The average severity grade of landmark distortion was 0.20 ± 0.50 in the tissue-engineered dermis group and 0.50 ± 0.71 in the artificial dermis group (*p* < 0.05). These findings indicate that tissue-engineered dermis grafts containing MAT are superior to artificial dermis grafts for facial reconstruction in terms of healing time, scar contraction, and landmark distortion severity. However, there was no significant difference in color mismatch between the two groups.

## 1. Introduction

Skin cancer is the most prevalent type of malignancy in humans, with its incidence rate increasing globally [1,2]. Due to its association with prolonged exposure to UV radiation, the faces of elderly individuals are especially susceptible to skin cancer. For optimal facial reconstruction in this population, it is essential to prioritize safe and minimally invasive techniques with the best possible aesthetic results.

Among options for facial reconstruction, local flap techniques are commonly favored due to their ability to integrate smoothly with surrounding tissues. However, local flaps might not always be viable when sufficient donor tissue is unavailable [3]. The invasive nature of flap surgery involving donor site flap creation and significant tissue undermining for successful transfer can cause visible scars on the face and impose considerable strain on patients, especially those in poor general health, such as elderly individuals. Additionally, flap transfers might alter aesthetic landmarks, especially near sensitive areas such as the eyes or nose. Other challenges include potential issues with blood supply to the flap or incomplete wound closure, which may result in less favorable outcomes [4]. Therefore, achieving optimal facial coverage with local flaps requires specialized expertise and experience.

Skin grafting is often used as an alternative. However, it presents several challenges. Key concerns include difficulties in achieving color match, a patchy appearance with visible borders, unpredictable pigmentation at the graft site, and possible complications at the donor site. Skin graft scars are especially prominent in individuals with darker-skinned patients, including Asian descent [5].

Advances in bioengineering have led to the development of artificial dermis as an alternative to autologous tissue reconstruction. Artificial dermis grafting offers a straightforward approach, eliminating extensive demands for autologous graft surgery and minimizing the risk of donor site morbidity. To meet the specific needs for reconstructing facial defects of elderly patients, our group has employed artificial dermis grafting and achieved acceptable outcomes. However, in some cases, this approach has resulted in conspicuous, unfavorable scars. To overcome limitations of artificial dermis alone, we have explored tissue-engineered dermis by combining autologous cultured dermal fibroblasts or adipose-derived stromal vascular fraction (SVF) cells with artificial dermis. This approach has shown promising results, achieving satisfactory outcomes without significant complications [6,7]. Despite these advancements, it is important to note that clinical applications involving cultured human fibroblasts or SVF cells require Food and Drug Administration (FDA) approval in addition to the time-intensive procedures needed for cell culture or isolation. Moreover, obtaining the required equipment, space, and specialized personnel for cell procurement can further complicate clinical use of these cells.

A recent advancement in regenerative medicine involves developing micronized adipose tissue (MAT) through mechanical dissolution instead of relying on enzyme-based methods. MAT consists of three primary components: cellular elements (including adipose-derived stem cells, fibroblasts, immune cells, and endothelial progenitor cells), extracellular matrices (ECMs), and cytokines. Both in vitro [8] and in vivo [9] studies on MAT grafts have demonstrated promising effects on protein synthesis, angiogenesis stimulation, and antioxidant activity. Leveraging the regenerative potential and composition of MAT, we have recently developed a tissue-engineered dermis graft using MAT to treat deep facial defects created by skin cancer resection.

This study aimed to evaluate scar outcomes following tissue-engineered dermis grafting with MAT and explore the potential of this technique for reconstructing deep facial defects.

## 2. Patients and Methods

This study protocol was approved by the Institutional Review Board (IRB) of the authors’ institution (IRB approval number: 2019GR0100). This study adhered to the principles of the Declaration of Helsinki. Informed consent was obtained from each patient.

Among skin cancer patients treated at our center between September 2022 and October 2023, those who received grafts of artificial dermis or tissue-engineered dermis containing MAT following skin cancer excision on the face with a follow-up period of more than one year were included. The difference between artificial dermis and tissue-engineered dermis is that the former does not contain MAT, whereas the latter does. The artificial dermis used in this study (INSUREGRAF^®^, Hyundai Bioland, Cheongju, Republic of Korea) is a highly porous, acellular native dermal matrix composed entirely of collagen derived from porcine skin. This structurally intact native collagen serves as a crucial component of the new extracellular matrix, facilitating cell migration and vascularization. Before selecting an artificial dermis or tissue-engineered dermis graft method using MAT, patients were provided with detailed information about the procedure, including its technique, advantages, and disadvantages. The final decision on the treatment method was made by the patients themselves. Cases were excluded if clinical photographs were unavailable or unsuitable for analysis. Patients with a history of other surgical procedures on the face were also excluded. Patient demographics including sex, age, cancer type, defect size, and re-epithelization time were collected from electronic medical records.

## 3. Surgical Technique

Skin cancer was excised with clinically tumor-free resection margins confirmed intraoperatively using frozen section biopsy. Defect areas were measured with a Visitrak Digital Wound Measurement System (Smith & Nephew, Hull, UK).

**Figure 1:**
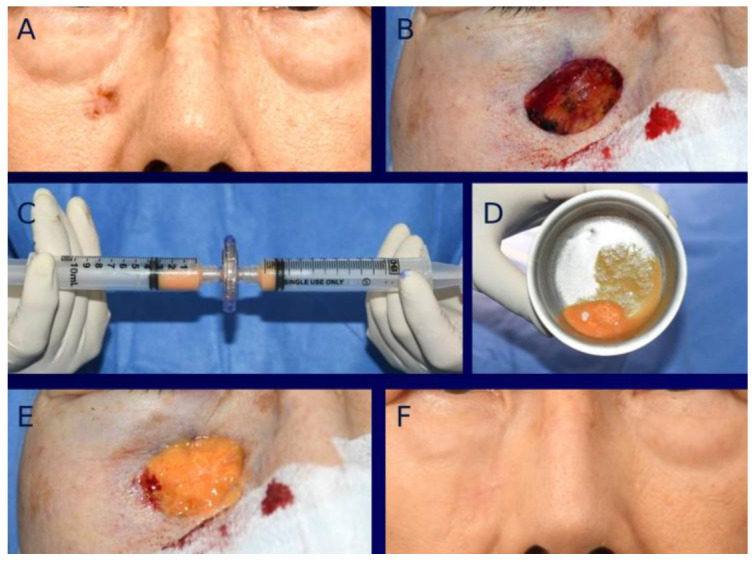
(**A**) A 78-year-old female presenting with basal cell carcinoma (BCC) in the right infraorbital region. (**B**) Intraoperative photograph showing facial defect following BCC resection. (**C**) Freshly harvested adipose tissue undergoing micronization, where the tissue is processed through a blade-edged micronizer filter. (**D**) Micronized adipose tissue (MAT) incorporated into artificial dermis to create a tissue-engineered dermis. (**E**) Intraoperative photograph immediately after grafting tissue-engineered dermis containing MAT. (**F**) Frontal view photograph taken at one year post-healing.

For tissue-engineered dermis grafting with MAT, abdominal adipose tissue was obtained via liposuction and then mechanically micronized using micronizer filters (Adinizer; BSLrest, Busan, Republic of Korea) in a descending order of pore size: first 1200 μm, then 600 μm, and finally 200 μm. An artificial dermis (INSUREGRAF^®^) was then tailored to fit the size and shape of the defect area. The MAT was evenly incorporated into the artificial dermis, creating a tissue-engineered dermis, which was subsequently grafted to the defect. Two or three layers of MAT-impregnated tissue-engineered dermis were utilized to align with the defect. Grafts were attached to the defect with the aid of fibrin glue (Baxter Healthcare Corp., Vienna, Austria). A meshed polyurethane film with a silicone adhesive (Mepitel; Mölnlycke Health Care, Gothenburg, Sweden) was applied as the primary dressing. A secondary layer of polyurethane foam (Mepilex; Mölnlycke Health Care) was then applied. The graft and foam were left in place for 5–7 days, with dressing replaced on a weekly basis (Figure 1).

**Figure 2:**
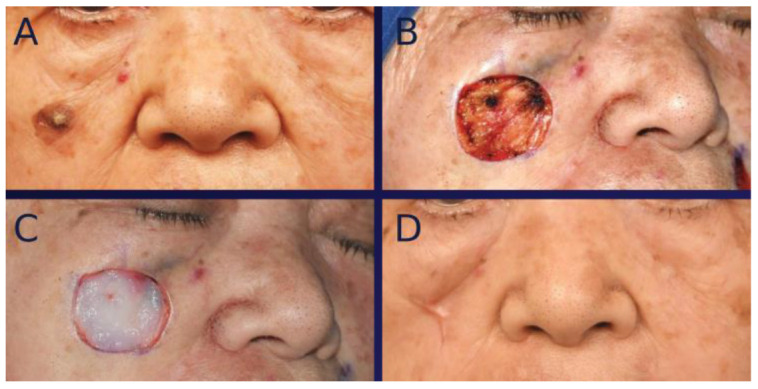
(**A**) An 83-year-old female presenting with basal cell carcinoma (BCC) in a similar location to the case in Figure 1. (**B**) Intraoperative photograph showing facial defect following BCC resection. (**C**) Intraoperative photograph immediately after grafting artificial dermis without micronized adipose tissue (MAT). (**D**) Frontal view photograph taken at one year post-healing. This case had a conspicuous scar with contracture.

For the artificial dermis graft, the MAT-free artificial dermis was applied topically in three to ten layers over the wound. All procedures, including wound care, were identical to those performed for tissue-engineered dermis graft (Figure 2).

## 4. Standardization of Clinical Photographs

All clinical photographs were taken by an experienced plastic surgeon with the patient in the Frankfort position. Patients were instructed to gaze directly at fixed points located two meters from the camera. The distance from the midpupil to the menton was used as a reference. Magnification was adjusted to ensure that this length remained consistent across each patient’s photographs.

## 5. Evaluation

The healing times of the two groups were compared by determining the number of days required to achieve complete epithelialization, defined as the absence of any discharge. All scar evaluations were conducted using patient photographs taken intraoperatively and 12 months post-healing.

### 5.1. Analysis of Color Mismatching

Patient photographs were imported into Photopea software (v.5.6; Photopea Inc., Prague, Czech Republic) and analyzed based on the Commission Internationale de l’Éclairage (CIE) color system, which maps color to coordinates L* (lightness), a* (red/green), and b* (yellow/blue). By selecting a specific point of the photograph, the L*, a*, and b* values at that location can be obtained. Using this system, objective differences in color matching were quantified by calculating the dE2000 score, with a higher dE2000 score indicating a more perceptible color difference between the two areas [10,11]. The detailed calculation follows the method described in Luo et al.’s article [12]. Briefly, this calculation can be automated using a pre-existing Excel package. All calculations were performed using the DeltaE2000 function from the ColorTools2.xla package (v.2.1.0; Edgardo Garcia, 2023, Buenos Aires, Argentina) [13].

**Figure 3:**
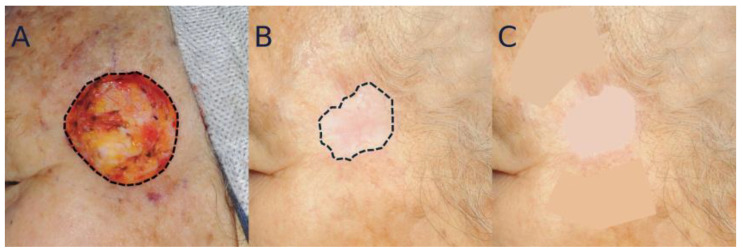
Analysis of color mismatch and scar contraction. (**A**) Intraoperative photograph of the temporal defect following BCC resection. The Lasso tool was used to define the boundaries of the defect. (**B**) Photograph taken one year after healing, where the Lasso tool was employed to outline the boundaries of the contracted scar, marking the area for the application of the Blur command. (**C**) Photograph post-application of the Blur command. The difference in color values between the average of two adjacent areas and the scar area was calculated.

The Lasso tool was used to outline the scar and two adjacent skin areas. These selected areas were then averaged using the Blur tool, and the Lab* color values were extracted from each area. The final colorimetric analysis was based on the average dE2000 score calculated between the scar and each comparison area (Figure 3).

### 5.2. Analysis of Scar Contraction

For quantitative analysis, the scar area outlined using the Lasso tool was measured at 12 months post-healing. After photograph standardization, the contracted area was determined by subtracting the scar area from the intraoperative defect area. The amount of scar contraction was then calculated as a percentage by dividing the contracted area by the intraoperative defect area (Figure 3). Surface areas, defined by predefined boundaries, were calculated using ImageJ software version 1.54 (National Institute of Health, Bethesda, MD, USA) [14].

For qualitative analysis, the severity of distortion of nearby landmarks was categorized into three grades (0, 1, 2). Grade 0 indicates cases where distortion is imperceptible to average human eyes. Grade 1 represents cases with minimal distortion, perceptible by human eyes but considered clinically insignificant. Grade 2 denotes marked distortion, resulting in a significantly disfiguring appearance. The grade was assessed by two blinded independent evaluators (plastic surgeons). Scores of 0, 1, and 2 were assigned for grades 0, 1, and 2, respectively. The average score determined by the two evaluators was used for data analysis.

Complications, including functional disturbances due to scar contracture and the development of hypertrophic scars or keloids, were also evaluated.

## 6. Statistical Analyses

Data are expressed as means ± standard deviations, and Mann–Whitney U tests were employed to compare defect area, healing time, distortion grade, and dE2000 score between two groups. Student’s *t*-tests were applied to analyze patients’ age and amount of scar contraction. Sex distribution, cancer subtypes, and cancer locations were analyzed using the chi-square test. Data were collected in Microsoft Excel (Microsoft Corp., Redmond, WA, USA). All analyses were performed using R software for Windows version 4.4.2 (the R Foundation for Statistical Computing, Vienna, Austria; http://www.r-project.org (accessed on 31 October 2024)). Analyzed data were processed to figures using the ggpubr R package (v.0.6.0; Kassambara, A. 2022, Marseille, France). All *p*-values were two-sided and statistical significance was set at *p* < 0.05.

## 7. Results

A total of 63 patients with 65 defects were included in this study, with two patients presenting two cancers each. Of these, 29 patients (14 males and 15 females; 30 defects) with a mean age of 71.4 ± 8.6 years were treated with tissue-engineered dermis grafts containing MAT. The remaining 34 patients (15 males and 19 females; 35 defects) with a mean age of 73.5 ± 10.0 years received artificial dermis grafts. In the tissue-engineered dermis group, 20 of 30 defects were due to basal cell carcinoma and 10 of 30 defects were due to squamous cell carcinoma. Defect locations included fifteen on the nose, six in the temporal region, five in the orbital region, and four in other areas. In the artificial dermis group, 24 of 35 defects were due to basal cell carcinoma and 11 were due to squamous cell carcinoma. Defect locations in this group included sixteen on the nose, six in the temporal region, six in the orbital region, and seven in other areas. Following skin cancer excision, the mean defect area was 3.7 ± 2.6 cm^2^ in the tissue-engineered dermis group and 3.4 ± 2.3 cm^2^ in the artificial dermis group. There was no statistically significant difference in age, sex, cancer type, defect location, or defect area between the two groups (Table 1).

All grafts adhered successfully to wound beds without any graft failures. Re-epithelialization time was 30.0 ± 4.3 days in the tissue-engineered dermis group and 34.3 ± 5.4 days in the artificial dermis group, showing a significant (*p* < 0.05) difference between the two.

**Figure 4:**
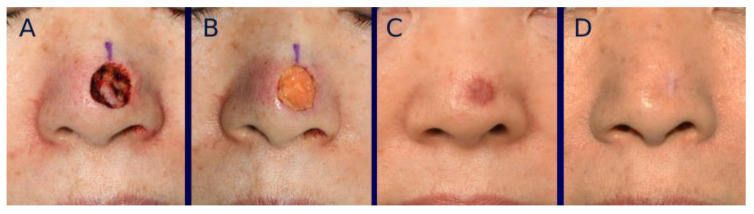
A 71-year-old female with basal cell carcinoma (BCC) on the nose. (**A**) Intraoperative photograph of the nasal defect after BCC resection. (**B**) Intraoperative photograph immediately after grafting tissue-engineered dermis containing micronized adipose tissue (MAT). (**C**) Photograph of the wound at 28 days post-grafting, showing complete healing with a prominent deep pink coloration. (**D**) Photographs taken at one year post-healing.

**Figure 5:**
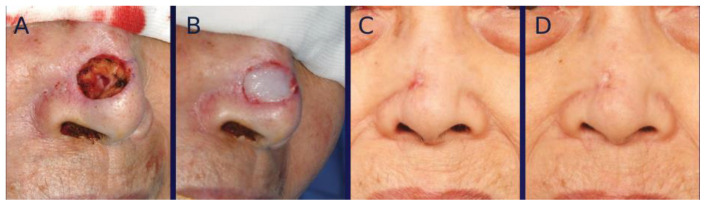
A 77-year-old male with basal cell carcinoma (BCC) located similarly to the case shown in Figure 4. (**A**) Intraoperative photograph of the nasal defect after BCC resection. (**B**) Intraoperative photograph immediately after grafting artificial dermis without micronized adipose tissue (MAT). (**C**) Photograph of the wound at 35 days post-grafting, showing complete healing with a prominent deep pink coloration. (**D**) Photographs taken at one year after healing, showing a less favorable scar appearance compared to the case in Figure 4.

For color mismatch analysis, a prominent deep pink color was noticeable in both groups at early post-healing stages, which gradually improved over time (Figure 4 and Figure 5). The average dE2000 score at one year post-healing was 4.9 ± 1.7 in the tissue-engineered dermis group and 5.1 ± 1.7 in the artificial dermis group, showing no significant (*p* = 0.57) difference between the two.

**Figure 6:**
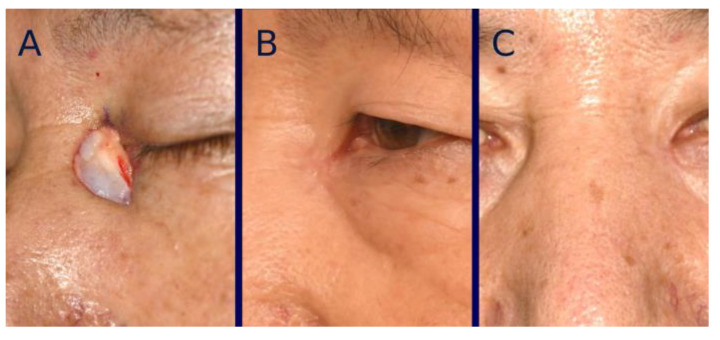
Representative case of Grade 0. (**A**) Intraoperative photograph immediately after grafting tissue-engineered dermis containing micronized adipose tissue (MAT). (**B**,**C**) Oblique and frontal view photographs taken at one year post-healing.

**Figure 7:**
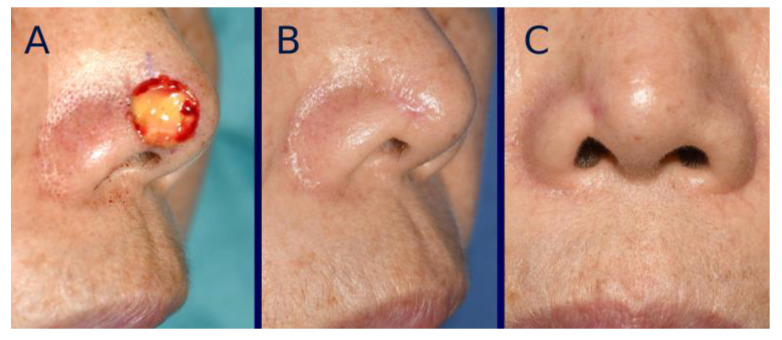
Representative case of Grade 1. (**A**) Intraoperative photograph immediately after grafting tissue-engineered dermis containing micronized adipose tissue (MAT). (**B**,**C**) Oblique and frontal view photographs taken at one year post-healing. Mild upward distortion of the patient’s right nostril apex was noted but deemed clinically insignificant.

**Figure 8:**
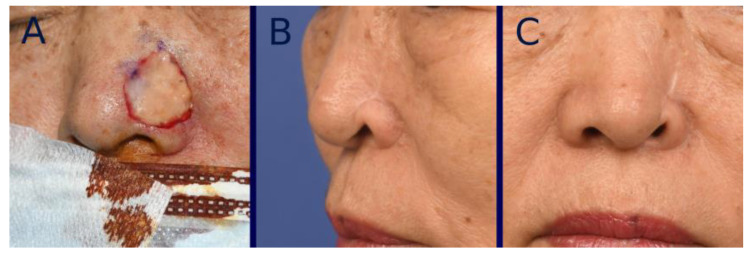
Representative case of Grade 2. (**A**) Intraoperative photograph immediately after grafting tissue-engineered dermis containing micronized adipose tissue (MAT). (**B**,**C**) Oblique and frontal view photographs taken at one year post-healing. Marked alar retraction was observed, resulting in a disfiguring appearance.

Scar contracture was also initially observed in many cases in both groups, although it gradually decreased over time. The extent of scar contraction at post-healing one year was 16.2 ± 12.3% in the tissue-engineered dermis group and 23.2 ± 12.8% in the artificial dermis group, showing a significant (*p* < 0.05) difference between the two. The average severity grade of landmark distortion at one year post-healing was 0.20 ± 0.50 in the tissue-engineered dermis group and 0.50 ± 0.71 in the artificial dermis group, showing a significant (*p* < 0.05) difference between the two (Figure 6, Figure 7 and Figure 8). Grade 0 distortion was noted in 83% (25/30) of cases in the tissue-engineered dermis group and 60% (21/35) of cases in the artificial dermis group. Grade 2 distortion was observed in one case in the tissue-engineered dermis group and four cases in the artificial dermis group, with all cases involving the lower nasal vault (Table 2).

All skin defects healed completely without complications such as hematoma, seroma, or wound infection. Functional complications related to tissue-engineered or artificial dermis grafting were not observed. There were no hypertrophic or keloid scars.

## 8. Discussion

Artificial dermis accelerates tissue granulation by promoting host cell and blood vessel migration, enabling rapid tissue replacement. Most commercial artificial dermis products contain collagen, a biocompatible protein that supports cellular recruitment, proliferation, and dermal regeneration [15,16]. Collagen is effective in aiding epidermal cell differentiation and migration. It may accelerate epidermal regeneration by increasing local growth factor concentrations [17]. Additionally, collagen helps control collagenase activity and extracellular matrix breakdown, which may reduce scar formation. However, based on our experience, this treatment can result in unfavorable scars in some cases. We hypothesized that incorporating patients’ autologous cells into the artificial dermis could improve scar quality. Thus, we conducted both animal and clinical studies to test this hypothesis. Our results confirmed positive effects of seeding cells into the artificial dermis [6,7,18].

Various studies have investigated effects of MAT on wound healing, reporting favorable and promising outcomes. However, clinical research on the impact of tissue-engineered dermis containing MAT on wound healing and scar quality remains lacking. To our knowledge, this study is the first to evaluate the efficacy and safety of MAT-containing tissue-engineered dermis in enhancing wound healing and scar quality for facial skin and soft tissue defects.

The encouraging outcomes of tissue-engineered dermis graft containing MAT could be attributed to the rich presence of essential wound-healing factors within the MAT. First, cellular constituents of MAT, including fibroblasts, endothelial cells, and ASCs, play a crucial role in promoting tissue regeneration and reducing wound contraction. In 2016, Ceserani et al. observed that MAT obtained through mechanical methods preserved angiogenic and anti-inflammatory properties in vitro, outperforming cultured ASCs [8]. MAT is regarded as a promising and safe alternative, circumventing complex regulatory challenges associated with enzymatic treatments and cell cultures. Second, ECM components and cytokines within the MAT construct can form a scaffold that supports robust tissue regeneration. Additionally, the three-dimensional (3D) architecture of tissue-engineered dermis with MAT can accelerate the volume-replacement effect, promoting faster healing by stimulating cell homing and paracrine signaling. In the present study, patients who received tissue-engineered dermis grafts demonstrated a faster wound healing rate and less scar contraction than those in the artificial dermis group (*p* < 0.05 for both).

The precise mechanism by which cellular components can reduce wound contraction is still not fully understood. Myofibroblasts are believed to play a key role in this process. Cells in the MAT may decrease levels of mast cells and myofibroblasts during the inflammatory phase through their immunosuppressive and anti-inflammatory effects [19]. Another possible explanation is that these cells can inhibit the migration of fibroblasts to the wound site. Migrating fibroblasts have the potential to differentiate into myofibroblasts, which are known to cause wound contraction. By preventing migration of fibroblasts capable of forming myofibroblasts from a wound bed, wound contraction might be minimized [20]. In addition to the role of myofibroblasts, ASCs in the MAT could significantly reduce wound contraction. ASCs might downregulate the expression of pro-fibrotic markers, such as α-smooth muscle actin and transforming growth factor beta1 [21,22], while upregulating antifibrotic factors like interferon-γ and matrix metalloproteinases, as well as pro-angiogenic factors such as vascular endothelial growth factor [21,23].

Before performing this study, we have reported promising results using MAT grafts alone without artificial dermis for treating diabetic foot ulcers and skin cancers [24,25]. In those studies, 3D bioprinting technology was used to consolidate and stabilize the MAT as it behaved similarly to a liquid state. Consequently, the preparation of MAT for grafting took over 50 minutes. Additionally, the process required a 3D bioprinter, a software program to transfer images to the printer, and personnel to operate the system. As an alternative, we recently developed a method to create tissue-engineered dermis by incorporating MAT into an artificial dermis. This approach is simpler, more time-efficient, and more cost-effective than using 3D bioprinting.

Facial scarring following reconstructive surgery can have a profound impact on patients’ psychological and social well-being [26,27]. Therefore, evaluating postoperative scars is a crucial factor when assessing reconstructive options. Consequently, assessing scars after surgery is essential for evaluating reconstructive approaches. Previous research has frequently relied on patient-reported outcomes or scar rating scales for aesthetic evaluation. However, these methods tend to be subjective. Moreover, characteristics such as pain, itching, pliability, thickness, and relief commonly examined in scar assessments are difficult to capture through photographs. Our study focused on assessing scar color and contraction, employing colorimetric and anthropometric tools to increase objectivity. Additionally, since facial aesthetics are influenced not only by the scar itself, but also by contracture-induced distortion of adjacent landmarks, we included this factor in our analysis.

The dE2000 score was initially designed as an industrial tool for evaluating color differences. It can quantify human perception of color variation. It has undergone substantial refinement for enhanced precision [10,11,13]. According to guidelines of the American Society for Testing and Materials, a dE2000 score under 4, between 4 and 8, or between 8 and 16 is deemed excellent, very good, or fair, respectively [10,28]. Based on these standards, the mean dE2000 score at 12 months post-healing indicated a ‘very good’ color match in both tissue-engineered and artificial dermis groups, showing no significant difference between the two.

Human skin color is influenced by four primary pigments: melanin (black/brown), carotene (yellow), oxygenated hemoglobin (red), and reduced hemoglobin (blue) [29]. In this study, oxygenated hemoglobin (red) is especially relevant as it reflects vasodilation and increased vascular permeability, which can raise oxygenated hemoglobin levels [30]. Vascular growth stimulated by tissue-engineered or artificial dermis grafting at the defect site contributed to early-stage scar redness post-healing. A randomized controlled trial by Bond et al. has demonstrated that scar redness usually diminishes around seven months post-healing [30]. Our findings support this, with scar color achieving a ‘very good’ rating within a year.

Only a few studies have objectively examined the extent of scar contraction following graft surgery in clinical settings. We found no clinical research quantifying scar contraction after tissue-engineered or artificial dermis grafting. To the best of our knowledge, our study is one of the first to provide an objective assessment of scar contraction following tissue-engineered dermis grafting. These findings could aid both patients and clinicians in understanding and anticipating outcomes after this treatment.

The use of tissue-engineered dermis with MAT for reconstructing deep facial defects offers several benefits, including simplicity, ease of implementation, and short operative time. It eliminates the need for major surgical interventions, reducing the overall surgical burden on patients. This makes the technique particularly advantageous for individuals in poor health, such as elderly patients, who may experience great stress from invasive graft or flap surgeries and tend to have slow wound healing. Although most of our patients were in their 70s, we observed no significant wound complications. Additionally, this straightforward technique can avoid donor site morbidity. It does not require specialized expertise either. In addition, it can preserve facial contours and reduce postoperative discomfort. Thus, it is ideal for patients requiring reconstruction at multiple sites. An added benefit of utilizing tissue-engineered dermis is that the graft closely resembles the adjacent skin, resulting in minimal color discrepancy. Epidermal restoration through epithelialization driven by migration and proliferation of nearby epidermal cells including melanocytes can achieve similar density and activity of melanocytes and their precursors within the graft’s epidermis, matching the adjacent skin [31].

This study has several limitations. It shares the inherent limitations of a retrospective design, highlighting the need for further well-structured studies to reach more definitive conclusions. Scar outcomes were assessed exclusively through photographs, which might have introduced discrepancies between the actual scar and its two-dimensional photographic depiction in terms of size and color. Additionally, the study cohort included only Korean patients. Since wound healing processes might vary among ethnic groups, these findings might not be directly applicable to other populations. Future research with a more ethnically diverse sample is necessary to improve the generalizability of these results. Nonetheless, our study holds value as it might be the first to report scar outcomes after using tissue-engineered dermis with MAT on the face with objective assessment tools.

## 9. Conclusions

Tissue-engineered dermis grafts containing MAT can provide better outcomes than artificial dermal grafts in terms of healing time and reduced scar contraction following facial skin cancer removal. However, color mismatch showed no significant difference between the two groups. Although further research is necessary to comprehensively assess advantages of this approach, the findings of this study suggest that tissue-engineered dermis grafting with MAT might be a safe and reliable option that delivers satisfactory results for reconstructing full-thickness skin and soft tissue defects on the face.

## Figures and Tables

**Table 1:** Baseline characteristics of the patients.

Group	AD (n = 35)	MAT (n = 30)	*p*-Value
Age (years)	73.5 ± 10.0	71.4 ± 8.6	0.362
Sex			
Male (%)	16 (45.7%)	15 (50.0%)	0.924
Female (%)	19 (54.3%)	15 (50.0%)	
Defect size (cm2)	3.4 ± 2.3	3.7 ± 2.6	0.807
Cancer subtype			1.000
Basal cell carcinoma	24 (68.6%)	20 (66.7%)	
Squamous cell carcinoma	11 (31.4%)	10 (33.3%)	
Location			0.906
Nose	16 (45.7%)	15 (50.0%)	
Orbit	6 (17.1%)	5 (16.7%)	
Temple	6 (17.1%)	6 (20.0%)	
Others	7 (20.0%)	4 (13.3%)	

AD—artificial dermis; MAT—micronized adipose tissue.

**Table 2:** Summary of the study outcomes.

Group	AD (n = 35)	MAT (n = 30)	*p*-Value
Healing time (days)	34.3 ± 5.4	30.0 ± 4.3	0.001
Scar contraction (%)	23.2 ± 12.8	16.2 ± 12.3	0.030
Landmark distortion (score)	0.50 ± 0.71	0.20 ± 0.50	0.043
Color mismatch (score)	5.1 ± 1.7	4.9 ± 1.7	0.567

AD—artificial dermis; MAT—micronized adipose tissue.

## Data Availability

The data presented in this article are not publicly available because the patients included in this study provided consent solely for academic publication in journals to contribute to medical advancements.
